# The Quality of Caregivers for the Elderly in Long-Term Care Institutions in Zhejiang Province, China

**DOI:** 10.3390/ijerph16122164

**Published:** 2019-06-19

**Authors:** Yuhang Zeng, Xiaoqian Hu, Yuanyuan Li, Xuemei Zhen, Yuxuan Gu, Xueshan Sun, Hengjin Dong

**Affiliations:** Center for Health Policy Studies, School of Public Health, Zhejiang University School of Medicine, 866 Yuhangtang Road, Hangzhou 310058, China; zengyuhang@zju.edu.cn (Y.Z.); huxiaoqian@zju.edu.cn (X.H.); sun89521@126.com (Y.L.); zhenxuemei@zju.edu.cn (X.Z.); guyuxuan@zju.edu.cn (Y.G.); sunxueshan@zju.edu.cn (X.S.)

**Keywords:** elderly care, caregivers, knowledge-attitude-practice, workforce, China

## Abstract

China is facing challenges in caring for older adults. This paper aimed to understand knowledge, attitude, and practice (KAP) regarding the quality of caregivers for the elderly in long-term care institutions in Zhejiang Province, and also to find related factors to improve the quality of caregivers. A cross-sectional survey was conducted from April to June 2016 in Zhejiang Province. In total, 84 caregivers were interviewed face-to-face with questionnaires on KAP towards elderly care. Multiple linear regression was used to find the related factors to KAP. A conceptual model was made to process path analysis among KAP and influencing factors using structural equation modeling. The study found that most caregivers in Zhejiang Province were middle-aged, female, and with a diploma below middle school. Many caregivers had not received any pre-employment training. Their salary was low although they undertook high-intensity work. Education and working years had a positive effect on knowledge and practice scores, and pre-employment training had a positive effect on knowledge and attitude scores. Knowledge and attitude regarding elderly care could positively affect elderly care practices. The quality of caregivers in Zhejiang Province was at a low level compared to developed countries. Continuous and regular elderly care training should be provided for caregivers to improve their elderly care knowledge and hence the quality of elderly care.

## 1. Introduction

China is facing unprecedented challenges in caring for older adults [1]. The proportion of the population aged 65 years and older is estimated to reach 22.6% by 2040, double that in 2010 (9%) [2]. In addition, older adults are at high risk for chronic diseases and become functionally disabled [3]. The increase in the number of elderly people, the increase in life expectancy and the decline in fertility rate have aggravated the overall burden to society to care for the aged. The old-age dependency ratio, which is the number of people aged 65 years or older for every 100 people aged 20 to 64, is expected to rise from 13 in 2010 to 45 in 2050 [4].

The traditional Chinese view is that the family members should take responsibility for taking care of the elderly as an expression of the Chinese cultural value of “filial piety” [5,6]. However, rapid demographic and socioeconomic shifts are weakening this system of family care [7]. The Chinese family size reduced due to three decades of the one-child family policy [5,8], resulting in a decreased number of available family caregivers [9]. In particular in urban areas, multigenerational families are being replaced by family structures with less potential for taking care of dependent older adults, such as the “4-2-1 family” (four grandparents, two parents, and one child) [1,10]. In addition, many old people choose to live apart from their adult children, leading to increasing numbers of “empty nest” families [7,8]. There are increasing numbers of adult children migrating to urban areas to seek jobs, leaving their parents behind in the countryside [4]. 

These factors have created a demand for formal long-term care (LTC). Thus, the Chinese government started to establish a three-tier care system: home-based care as the foundation; community-based services as a support; and institutional care as a supplement [4]. Nowadays, both the elderly themselves and their families are increasingly accepting of institutional care [11]. The number of older adults aged above 65 years living in nursing homes increased from 1.03 million in 2004 to 3.20 million in 2014 [12]. However, nearly 12 million elderly people are willing to live in nursing homes, far exceeding the supply capacity [13]. What is more, it is usually older adults with cognitive and physical function impairment who are more likely to use LTC with a great need for daily and health care [14,15].

As a developing industry, Chinese LTC institutions are faced with the severe challenge of a poorly prepared workforce in terms of both quantity and quality [16]. On the one hand, the long working hours, heavy workload, low wages and emotional stress results in severe staff shortage and a high staff turnover [13]. On the other hand, many researchers have reported that Chinese LTC facilities lack professional and qualified caregivers [11,17]. Most caregivers in institutions were 40–60-year-old females with a diploma below junior high school [16]. The majority of caregivers in urban areas were rural migrants. In addition, they had limited professional knowledge about elderly care [16,17]. Less than one-third of caregivers have received formal training according to the most optimistic estimation [11]. Chinese nursing homes had few qualification standards for staff training compared with other countries [16]. Thus, the workforce, which is lacking qualified and professional caregivers, could not meet the urgent needs of the elderly for formal and professional LTC.

**Figure 1:**
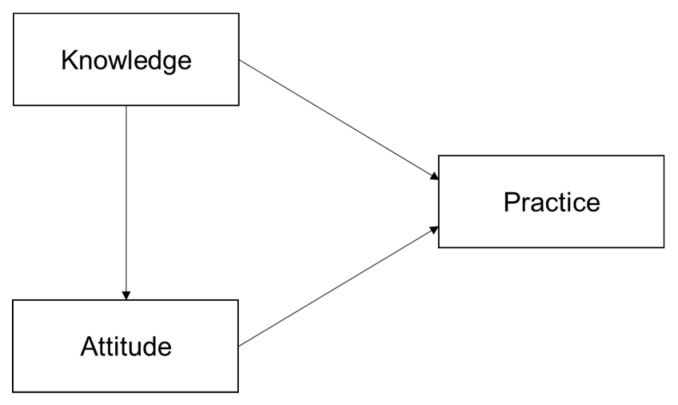
Conceptual model of knowledge, attitude and practice.

Behavior change theories [18] believe that individuals with sufficient knowledge and positive attitudes could result in good practice. Knowledge and attitude are the driving forces for the change in behavior, and only when people understand health-related knowledge and establish a positive attitude is it possible to form correct behavior [19]. Researchers found that nursing care offered by care staff in the LTC institutions could be affected by their knowledge and attitude, which would eventually influence the quality of the health wellbeing of the older adults. A Sweden study found that nursing staff with no formal competence perceived higher workload, more communication obstacles and more stress than their colleagues with formal competence [20]. A study in the United States found that after training certified nurse aides in mouth care, the oral health of residents was improved [21]. However, there are seldom studies in China to explore the potential pathways among knowledge, attitude, and practice of elderly care among caregivers. Thus, we made the following hypotheses and a conceptual model (Figure 1) to explore their associations:

**H1.**  *Elderly care knowledge affects attitude towards elderly care.*

**H2.**  *Elderly care knowledge affects elderly care practices.*

**H3.**  *Attitudes towards elderly care affects elderly care practices.*

This study focused on investigating the quality of caregivers for the elderly, namely formal care staff in LTC institutions whose work consists of taking care of elderly residents. Our study had three objectives: First, to investigate the demographic characteristics, elderly care knowledge, attitude towards elderly care and elderly care practices among caregivers in Zhejiang Province. Second, to find influencing factors related to their knowledge, attitude and practice. Third, to explore the associations between knowledge, attitude and practice regarding elderly care and related factors. Finally, to provide suggestions to improve the quality of caregivers in LTC facilities and eventually improve the quality of elderly care offered by caregivers.

## 2. Materials and Methods

### 2.1. Data Sources

The data used in this study was from a cross-sectional survey conducted from April to June 2016 in Zhejiang province, China. We adopted a multi-stage stratified cluster random sampling method. In the first stage, according to cluster analysis from the perspective of Gross Domestic Product (GDP), we randomly selected Hangzhou city and Jinhua city to represent developed and underdeveloped areas, respectively. In the second stage, Xihu district in Hangzhou and Lanxi county in Jinhua city were randomly selected to represent urban and rural areas, respectively. In the third stage, in line with the proportion of different types of LTC institutions, we randomly selected two public-owned institutions and two private-owned institutions in Xihu district; and two public-owned institutions and four private-owned institutions in Lanxi county. All caregivers in the selected institutions were interviewed face-to-face by trained interviewers. Interviewers would explain the research purpose and all questions in the languages that respondents were fluent in, in case some respondents were literate and not able to read. Written informed consent for all participants in the survey was obtained before the survey. The Ethics Committee of the School of Public Health, Zhejiang University approved this study (No. ZGL20150134).

### 2.2. Data Collection

According to a literature review of previous comparable studies [22,23,24] and the “National Occupational Standards for Old-Age Caregivers” [25], which was issued by the Chinese Ministry of Civil Affairs and shown to have high reliability and validity, we developed and adapted a structured questionnaire to assess caregivers’ knowledge, attitude, and practice regarding elderly care.

In order to ensure validity, the questionnaire was discussed by experts from the health administrative department, civil affairs department and universities to evaluate its design, content relevance, appropriateness and readability. We modified the questionnaire based on their comments. A pilot survey was conducted in two LTC institutions that were not included in the formal survey to assess the applicability of the questionnaire. According to the results of the pilot survey, we modified questions that were ambiguous or difficult to understand. During the formal survey, a fieldwork supervisor was arranged to monitor the investigation process and conduct quality control. The quality assurance measures for this survey involved training the interviewers and checking the validity of the collected data. We calculated the Cronbach’s alpha value to assess the reliability of the questionnaire—the recommended acceptable value was larger than 0.70 [26]. The Cronbach’s alpha value of our questionnaire was 0.76, indicating acceptable internal consistency.

The questionnaire involved four sections. The first section covered sociodemographic information and working situation. The sociodemographic information contained age, gender, residence (native or not), education, and monthly salary. The working situation included working years, pre-employment training (to ask whether the respondents have attended any pre-employment training), on-job training times (to ask about the number of times respondents have attended training after they got the job) and average daily working hours.

The second section examined participants’ knowledge on elderly care. It was comprised of eight domains and twenty-three items. The eight domains evaluated knowledge about elderly diet, dementia, diabetes, chronic bronchitis, high blood pressure, bedsores, medication and elderly psychology. Participants were asked to answer questions and judge whether statements were true or false. Each correct response scored 1 point and an incorrect response or “don’t know” scored 0 points. The total score ranged from 0 to a maximum of 23—a higher score indicating that the participants had better knowledge on elderly care.

The third section examined participants’ attitudes towards elderly care. The attitude section contained thirteen questions about job satisfaction, job willingness and disease care. Participants were asked to rate every question based on a 5-point Likert scale of 1 (strongly disagree) to 5 (strongly agree)—the higher point represented by the more positive attitude. The total score ranged from 13 to a maximum of 65.

The fourth section examined participants’ elderly care practices. The practice section involved seven questions to evaluate whether the participants have completed these seven physical and mental nursing practices. Each question in this section had two answers—whether they did it or not. The participants could get one score for every practice they completed. The total score ranged from 0 to a maximum of 7.

### 2.3. Statistical Analyses

We used K-Score, A-Score and P-Score as indicators to show the score of the knowledge, attitude and practice section, respectively. We firstly made descriptive analyses to evaluate the basic situation—the K-score, A-score, P-score of caregivers in Zhejiang Province. Then, we used multiple-linear regression (stepwise) to explore the possible factors influencing knowledge, attitude and practice. Next, based on the conceptual model of knowledge, attitude and practice (Figure 1) and influencing factors related to KAP, we conducted a model to process path analysis among potential factors and KAP using structural equation modeling (SEM). SEM, a multivariate statistical method, was used in many fields of research to explore associations between variables [27,28,29]. Maximum likelihood was used to estimate the model. The goodness of fit index (GFI), comparative fit index (CFI), normed fit index (NFI), and root mean squared error of approximation (RMSEA) were used to evaluate the model fit. If the GFI, CFI and NFI were larger than 0.90 and RMSEA was less than 0.06, the model fit would be acceptable [30,31]. Descriptive analyses and multiple-linear regression were performed using the IBM^®^ SPSS^®^ Statistics ver. 20.0 (IBM, New York, NY, USA). Structural equation modeling was carried out using AMOS ver. 21.0 (IBM, New York, NY, USA).

## 3. Results

### 3.1. Socio-Demographic Characteristics

Eighty-four caregivers were interviewed. Table 1 showed that the vast majority of the caregivers were middle-aged females with an average age of 50.8 years old—even the oldest caregiver was 72.0 years old. Most of the caregivers only had a diploma below middle school, and a quarter of them were illiterate. In addition, their salary was below ¥ 4000 (about US$580) per month. Approximately 65% of them received no training before employment. In addition, nearly 20% of the respondents did not receive any on-job training. The average daily working time was nearly 15 h, much longer than the legal working hours (8 h).

### 3.2. Knowledge on Elderly Care

The mean knowledge score of the respondents is 17.44 ± 3.34 (the full score is 23). Among all the respondents, the highest score is 23 and the lowest score is six. Table 2 summarized the results for each question in the knowledge section. A majority of respondents have good knowledge on elderly care with regard to diet, high blood pressure, bedsores and medication. However, poorer knowledge was exhibited on the care of dementia, diabetes and psychological problems. Only a quarter of respondents were aware that older adults with dementia would exhibit consistent memory loss.

### 3.3. Attitude towards Elderly Care

The mean Attitude score of the respondents is 48.15 ± 3.71 (the full score is 65). Among all the respondents, the highest score is 60 and the lowest score is 38. Table 3 summarizes the results for each question in the attitude section. Among the average score of all questions, the average scores of satisfaction on work time and salary were lower. On the aspect of attitude towards disease care, the respondents showed moderate confidence in a range of care activities.

### 3.4. Practice Regarding Elderly Care

The mean practice score of the respondents is 5.58 ± 1.36 (the full score is seven). Among all the respondents, the highest score is seven and the lowest score is one. Table 4 summarizes the results for each question in the practice section. Most respondents have good practices regarding elderly care, especially on room cleaning.

### 3.5. Factors Influencing Knowledge, Attitude and Practice

From the results of the multiple linear regression analyses shown in Table 5, we found that working years, education and pre-employment training had a significant positive influence on respondents’ knowledge on elderly care. Pre-employment training and monthly salary positively influenced respondents’ attitude towards elderly care, while age had a significant negative influence. On-job training times, education and working years all positively influenced practice on elderly care.

### 3.6. Pathways among Knowledge, Attitude and Practice

**Figure 2:**
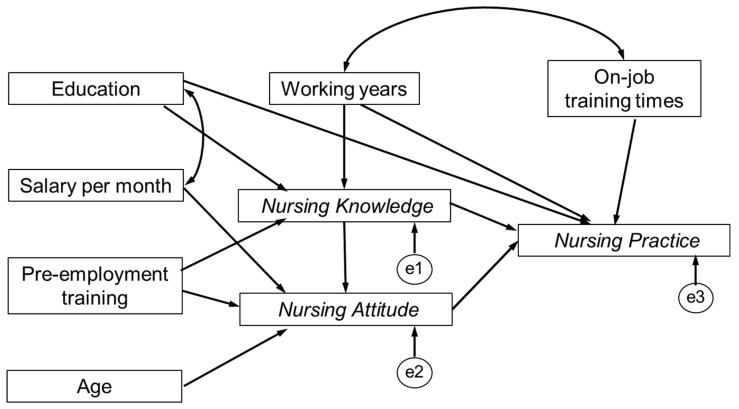
First-round model of knowledge, attitude, and practice regarding elderly care.

Based on the conceptual model (Figure 1) and related factors that we found from a multiple linear regression model, we conducted a structural equation model to explore the potential pathways among the knowledge, attitude and practice dimensions and related factors (Figure 2). The maximum likelihood was used to estimate the model. However, the first-round model fit was not acceptable. The CFI, GFI, NFI, RMSEA were 0.769, 0.546, 0.526, 0.249, respectively, which did not meet the acceptable criterion.

**Figure 3:**
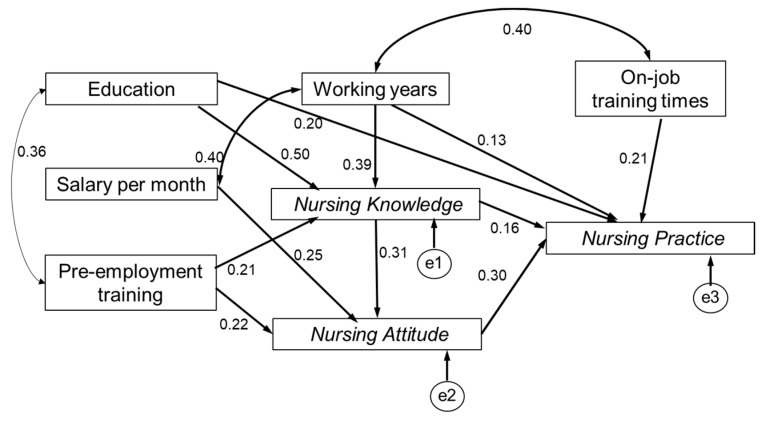
A structural equation model of knowledge, attitude and practice regarding elderly care.

Thus, we modified the first-round model according to model fit indices. Figure 3 presented the final model of pathways among the three dimensions and influencing factors. The CFI, GFI, and NFI were 0.977, 0.948, 0.907, respectively, which was larger than 0.90, indicating a good fit. The RMSEA was 0.058 and was considered a good fit if below 0.06. Based on the indexes, the final model had an acceptable fit. Knowledge had positive effects on attitude and practice, which sustained the first hypothesis (H1) and second hypothesis (H2). Attitude had a positive effect on practice, which sustained the third hypothesis (H3).

## 4. Discussion

In this study, we used cross-sectional data to investigate the quality of caregivers in LTC facilities in Zhejiang Province. We found that most of the caregivers were middle-aged females with diplomas below middle school. There were many respondents who had not received any pre-job training. Their work times were long while their salaries were low. Their average knowledge, attitude and practice were 17.11 ± 3.34, 48.15 ± 3.71, and 5.58 ± 1.36, (full score was 23, 65 and 7), respectively. Education and working years had a positive effect on the knowledge and practice scores, pre-job training had a positive effect on the attitude score. We also found that elderly care knowledge and attitude towards elderly care could positively affect caregivers’ elderly care practices.

An obvious gender disproportion, as most caregivers were female, was found in this research. This phenomenon was similar to other studies [17,32,33]. A study in Chengdu reported that 73% of direct caregivers were female [17]. An Australian study also found that women make up over 60% of the total caregivers [33], although female caregivers may not be physically strong enough to care of the elderly, for example, to help male bedridden residents turn over [32]. This problem may weaken the quality of care, and thus call for a larger workforce of male caregivers.

The caregivers in LTC facilities in Zhejiang Province had an average age of 50.8 years old. Most of the caregivers received education below junior high school. A majority of the caregivers began work without pre-employment training and qualified certification. Compared with other developed countries, the quality of caregivers in Zhejiang Province was poorer. In the United States, nursing staff were registered nurses, certified nursing assistants and nurse’s aides [17]. An American study using a U.S. National Nursing Assistant Study reported that nursing assistants had an average age of 37.0 years old and most of them had high school diplomas [16]. Henna found that two-thirds of the caregivers in Sweden nursing homes were under 50 years old. Meanwhile, licensed practical nurses constituted the largest group of caregivers [34]. An Australian study showed that caregivers had an average age of 44.7 years old. The majority of them had post-high school diplomas [35]. Our study found that age had a negative effect on the attitude of caregivers, while education had a positive effect on elderly care knowledge and practice. Similarly, much research indicated that high-quality caregivers were associated with the improved care they offered for elderly residents in LTC facilities [16,20,36]. Therefore, the quality of caregivers in Zhejiang Province needs to be improved in order to provide a better service for older adults. The government and administers of LTC facilities should make great efforts to attract high-quality and skilled younger caregivers.

We found that most respondents did not have sufficient pre- or post-employment training. In particular, they were short of knowledge on common disease care, especially dementia and diabetes. What is more, we confirmed that elderly care knowledge had positive effects on attitude and practice regarding elderly care. Thus, it was difficult for caregivers without sufficient elderly care knowledge to provide high-quality elderly care. In this study, we found that pre-employment training had a positive effect on nursing knowledge and attitude. On-job training times also had a positive effect on nursing practice. These findings highlight the importance of regular training for caregivers, which is consistent with previous research [11,16,21]. Although in China, the “National Occupational Standards for Old-Age Caregivers” promulgated in 2002 required that the basic requirements for elderly caregivers included a middle school education diploma and at least 180 h of training, it is widely believed that the number of caregivers who actually meet these basic requirements is very small [11]. However, in developed countries, caregivers must meet some standards before they start working. In the United States, the federal government mandates that all caregivers must receive 75 h of training before being qualified for working in Medicaid/ Medicare certified nursing homes [37]. Germany requests that at least a half the care staff in a nursing home are Registered Nurses who have had 3 years of geriatric training. The Organization for Economic Co-operation and Development ( OECD) demonstrated that certificates and training are important components of staff quality [38]. Thus, basic health qualification is supposed to be a compulsory requirement for caregivers [39]. Chinese policy makers should prioritize training initiatives through issuing national regulatory frameworks to build a professionalized LTC workforce.

There are two limitations in our study. First, we explored the pathways among socio-demographic factors, knowledge, attitude and practice based on 84 caregivers. If more caregivers were enrolled in this study, the results would become more convincing. Due to the pathways that have been found, the data we used is suitable. Second, the practice section in the questionnaire was evaluated subjectively by the caregivers themselves, which may result in bias. We will try to adopt other methods to improve the accuracy, such as observation by investigators.

## 5. Conclusions

In conclusion, the quality of caregivers for the elderly in LTC institutions in Zhejiang Province was at a low level. Most of the caregivers were middle-aged females without sufficient knowledge and training on elderly care. Because of the positive effect of knowledge and training on elderly care practice, Chinese policy makers should pay more attention to conduct and strengthen professional training for caregivers [39]. Meanwhile, a complete set of laws and regulations on improving the quality of caregivers should be established and executed compulsorily [17]. Continuous and regular elderly care training should be provided for caregivers to improve their elderly care knowledge and hence the quality of elderly care.

## Figures and Tables

**Table 1:** Socio-demographic characteristics of respondents (*n* = 84).

Variables	Number
Sociodemographic	
Age	
<40	7 (8.3)
40–49	21 (25.0)
50–59	46 (54.8)
>60	10 (11.9)
Gender	
Male	6 (7.1)
Female	78 (92.9)
Residence	
Native	74 (88.1)
Non-native	10 (11.9)
Education	
Illiterate	21 (25.0)
Primary school	20 (23.8)
Middle school	30 (35.7)
High school or higher	13 (15.5)
Monthly salary (¥)	
≤2500	11 (13.1)
2501~3000	26 (31.0)
3001~4000	45 (53.6)
4001~5000	2 (2.4)
≥5001	0
Working situation	
Working years	
<1 year	17 (20.2)
1~3 years	28 (33.3)
3~5 years	20 (23.8)
> 5 years	19 (22.6)
Pre-employment training	
Yes	29 (34.5)
No	55 (65.5)
On-job training times	
0	14 (16.7)
1	7 (8.3)
2~4	38 (45.2)
5~8	18 (21.4)
>9	7 (8.4)
Average working hours per day	14.96 ± 5.77

*Note.* Data are presented as mean ± standard deviation or *n* (%). Percentages may not add up to exactly 100 percent, owing to rounding off.

**Table 2:** Respondents’ knowledge on elderly care.

Category	Question or Statement	Correct Response, *n* (%)
Diet	Do the elderly need adequate water between meals?	81 (96.4)
	It is better for the elderly to have more meals and eat less at every meal.	75 (89.3)
	The elderly should drink lots of water quickly at once.	78 (92.9)
Dementia	The elderly with dementia would not exhibit consistent memory loss or forget their family members.	21 (25.0)
	The elderly with dementia would not exhibit incontinence.	78 (92.9)
	The elderly with dementia would experience hallucinations in the early stage of the disease.	54 (64.3)
	The elderly with dementia should not be allowed to walk around casually.	37 (44.0)
	We should often communicate with the elderly with dementia to help alleviate their symptoms.	80 (95.2)
Diabetes	When the elderly with diabetes suffer from dizziness, fatigue and other conditions when they go out, we should immediately hold them steady to avoid them falling down.	69 (82.1)
	It is normal for the elderly with diabetes to suffer from dizziness and fatigue, we do not need to undertake any action.	64 (76.2)
	We should do foot care for the elderly with diabetes every week to prevent foot complications.	37 (44.0)
	Elderly with diabetes need no special treatment in terms of diet, just be consistent with other elderly people.	33 (39.3)
Chronic bronchitis	We should change the clothes of the elderly with chronic bronchitis according to the temperature to prevent the disease from getting worse.	72 (85.7)
	In the more severe smog weather, the elderly with chronic bronchitis should be urged to exercise normally and stay healthy.	50 (59.5)
High blood pressure	For the elderly with high blood pressure, they should remain seated for long periods.	55 (65.5)
	Do the elderly with high blood pressure need to avoid salty, spicy food in their diets?	74 (88.1)
	For the elderly with high blood pressure, they should ensure reasonable rest time and appropriate physical exercise.	79 (94.0)
Bedsore	Bedsores occur mainly because of long-term bed rest.	68 (81.0)
	For the long-term bedridden elderly, they should be turned over every 2 h to observe whether their skin is red or not.	79 (94.0)
Medication	In the process of assisting the elderly to take medicine, they should be helped to a comfortable position and then given medicine.	79 (94.0)
	We can leave immediately after helping the elderly take medicine.	62 (73.8)
Psychological problems	It is normal for the elderly to have psychological problems, so no intervention is needed.	32 (38.1)
	We should properly communicate with the elderly for psychological care.	80 (95.2)

Note: Knowledge was assessed by giving one score to each correct answer.

**Table 3:** Respondents’ attitude on elderly care.

Category	Question	Average Score (± SD)
Job satisfaction	Would you like to receive further knowledge and skills training?	3.36 ± 0.93
	Are you satisfied with your current working hours?	3.17 ± 0.69
	Are you satisfied with your current salary?	3.07 ± 0.77
Disease care	Are you confident that you can take care of the elderly with dementia?	3.94 ± 0.39
	Are you confident that you can take care of the elderly with diabetes?	3.78 ± 0.52
	Are you confident that you can take care of the elderly with chronic bronchitis?	3.92 ± 0.35
	Are you confident that you can take care of the elderly with high blood pressure?	4.00 ± 0.27
	Are you confident that you can take care of bedridden elderly?	3.92 ± 0.44
	Do you think it is important to supervise the elderly to take their medicine on time?	4.19 ± 0.42
	Do you think that the psychological care of the elderly is important?	4.12 ± 0.50
Job willingness	Do you care what other people think of your work in elderly care?	3.36 ± 0.87
	Are you willing to work in elderly care for a long time?	3.75 ± 0.58
	Do you like the current work in elderly care?	3.60 ± 0.58

Note: Attitude was assessed by giving 1 (strongly disagree) to 5 (strongly agree) points. A higher score represents a more positive attitude. SD = standard deviation.

**Table 4:** Respondents’ practice on elderly care.

Question	Practice Rate, *n* (%)
Have you made any suggestions to the canteen in the nursing home about proper diets for the elderly?	30 (35.7)
Do you often clean the living environment of the elderly?	83 (98.8)
Are you concerned about the disease situation of the elderly?	77 (91.7)
Do you often pay attention to the behavior and activity of the elderly?	66 (78.6)
Have you supervised the elderly to take medicine on time?	78 (92.9)
Have you paid attention to the emotional changes of the elderly?	67 (79.8)
Do you often communicate with the elderly and provide psychological help?	68 (81.0)

**Table 5:** Factors influencing knowledge, attitude, and practice.

Variables	b	SE	b’	*p*-Value
Knowledge				
Intercept	9.305	1.081	-	<0.001
Education	1.275	0.219	0.521	<0.001
Working years	0.944	0.188	0.418	<0.001
Pre-employment training	1.528	0.608	0.219	0.014
R^2^ = 0.482				
Attitude				
Intercept	43.529	2.938	-	<0.001
Pre-employment training	2.033	0.801	0.262	0.013
Salary per month	1.571	0.440	0.355	0.001
Age	−0.090	0.045	−0.211	0.048
R^2^ = 0.262				
Practice				
Intercept	2.75	0.533	-	<0.001
On-job training times	0.263	0.119	0.237	0.031
Education	0.358	0.097	0.361	<0.001
Working years	0.266	0.101	0.290	0.010
R^2^ = 0.279				

Note: SE = standard error.
